# New or Blossoming Hemorrhagic Contusions After Decompressive Craniectomy in Traumatic Brain Injury: Analysis of Risk Factors

**DOI:** 10.3389/fneur.2018.01186

**Published:** 2019-01-15

**Authors:** Davide Nasi, Lucia di Somma, Maurizio Gladi, Elisa Moriconi, Massimo Scerrati, Maurizio Iacoangeli, Mauro Dobran

**Affiliations:** Department of Neurosurgery, Umberto I General Hospital, Università Politecnica delle Marche, Ancona, Italy

**Keywords:** decompressive craniectomy, traumatic brain injury, hemorrhagic contusion, expansion of hemorrhagic contusions, acute subdural hematoma

## Abstract

**Background:** The development or expansion of a cerebral hemorrhagic contusion after decompressive craniectomy (DC) for traumatic brain injury (TBI) occurs commonly and it can result in an unfavorable outcome. However, risk factors predicting contusion expansion after DC are still uncertain. The aim of this study was to identify the factors associated with the growth or expansion of hemorrhagic contusion after DC in TBI. Then we evaluated the impact of contusion progression on outcome.

**Methods:** We collected the data of patients treated with DC for TBI in our Center. Then we analyzed the risk factors associated with the growth or expansion of a hemorrhagic contusion after DC.

**Results:** 182 patients (149 males and 41 females) were included in this study. Hemorrhagic contusions were detected on the initial CT scan or in the last CT scan before surgery in 103 out of 182 patients. New or blossoming hemorrhagic contusions were registered after DC in 47 patients out of 182 (25.82%). At multivariate analysis, only the presence of an acute subdural hematoma (*p* = 0.0076) and a total volume of contusions >20 cc before DC (*p* = < 0.0001) were significantly associated with blossoming contusions. The total volume of contusions before DC resulted to have higher accuracy and ability to predict postoperative blossoming of contusion with strong statistical significance rather than the presence of acute subdural hematoma (these risk factors presented respectively an area under the curve [AUC] of 0.896 vs. 0.595; *P* < 0.001). Patients with blossoming contusions presented an unfavorable outcome compared to patients without contusion progression (*p* < 0.0185).

**Conclusions:** The presence of an acute subdural hematoma was associated with an increasing rate of new or expanded hemorrhagic contusions after DC. The total volume of hemorrhagic contusions > 20 cc before surgery was an independent and extremely accurate predictive radiological sign of contusion blossoming in decompressed patients for severe TBI. After DC, the patients who develop new or expanding contusions presented an increased risk for unfavorable outcome.

## Introduction

The expansion of a cerebral hemorrhagic contusion after TBI occurs commonly and it is a widely studied phenomenon (1). Several series have reported a rate of progression of hemorrhagic contusions ranging approximately from 38 to 59% of cases (1, 2). In the last 20 years, the use of decompressive craniectomy (DC) for the treatment of uncontrollable high intracranial pressure (ICP) after TBI (3–11) has gradually increased. Indeed, the DC increases brain compliance and reduces ICP in presence of diffuse cerebral edema or intracranial hematomas (5–11). However, several authors reported that the change in pressure dynamics after DC can lead to the relief of the tamponade effect allowing the growth and/or progression of a hemorrhagic contusions (12–14).

A recent review about the complications of DC (12) reported a rate of expansion of hemorrhagic contusions after DC of 12.6% of adult patients and Flint et al. (14) have suggested that the occurrence of expansion of hemorrhagic contusions was associated with unfavorable outcome after DC. For these reasons, the recognition of risk factors associated with the growth or expansion of hemorrhagic contusion can improve the management of decompressed patients, identifying a subgroup of patients who might benefit from an early post-operative CT scan and from several potential therapeutic maneuvers minimizing hemorrhagic complications of DC.

However, few studies investigated factors associated with new or expanding contusions following DC. The aim of our study was to identify the risk factors associated with the growth or expansion of hemorrhagic contusion after DC in TBI. Then we evaluated the impact of contusion progression on outcome.

## Materials and Methods

We included in this retrospective study all patients treated with DC for TBI in our Department from January 2003 to December 2011.

The protocol of our Hospital for the management of severe TBI, which includes indication for DC, was based on current literature and on the Brain Trauma Foundation guidelines for management of intracranial pressure (ICP) following traumatic brain injury, fourth edition (3).

The management objectives are ICP ≤ 20–25 mmHg and cerebral perfusion pressure (CPP) ≥60 mmHg. ICP was monitored in unconscious patients with mass lesions potentially evolutive or edema with intraventricular drainage catheter or intraparenchymal probes. Patients with large epidural/subdural haematomas and contusions with significant mass effect underwent to surgery and in case of introperative massive brain swelling a primary decompressive craniectomy was performed. In the other patients, head elevation, sedation with propofol and analgesics infusion and mannitol were used to control ICP value. If these actions failed to control elevated ICP, thiopental infusion was started. Finally, DC was considered a last-tier treatment in case of persistent high ICP after barbiturates administration or adverse effects of thiopental. Usually we used an unilateral DC if there was a shift of the midline or one dilated pupil. Bifrontal DC were performed with the posterior limit at the coronal sutures. in patient with diffuse edema or bifrontal contusions.

Unilateral DC of at least 15 cm diameter were performed with the medial limit at least 2–2.5 cm lateral to the midline.

Bifrontal DC was performed with this technique in all cases: a large bicoronal incision was made and a large bifronto-temporal craniotomy was performed about 1–3 cm behind the coronal suture and including the bone over the superior sagittal sinus. Then anterior portion of the sagittal sinus is ligated and divided between the stitches and finally the falx was divided completely to achieve a maximal decompression.

All cases underwent expansive duraplasty with an allograft (lyophilized bovine pericardium).

From our analysis, we excluded patients with antiplatelet or warfarin use. Furthermore, all patients with coagulopathies already known before the trauma were excluded from the study. Data on blood tests on coagulation were not collected.

The primary study outcome focused on the development or expansion of a hemorrhagic contusion after DC.

Baseline characteristics (age, sex, cause of TBI, admission Glasgow Coma Scale score (GCS), admission pupils' reactivity, extracranial injury and Rotterdam score) DC were recorded and analyzed (Table 1).

**Table 1:** Baseline characteristics of 190 patients with severe traumatic brain injury undergoing decompressive craniectomy.

**Variabile**	***N*°**	**(%)**
Total number of patiens	190	
Mean age (years)	50	
Range	14–86	
**SEX**		
Male	115	(60.52%)
Female	75	(39.48%)
**CAUSE OF TBI**		
Road traffic accidents	111	(58.4%)
Falls	75	(39.6%)
Altro	4	(2%)
**GCS AT ADMISSION**		
3–5	129	(67.9%)
6–8	59	(31.1%)
>8	2	(1%)
**PUPILS REACTIVITY TO THE LIGHT**		
Yes	156	(82.1%)
No	34	(17.9%)
**EXTRACRANIAL INJURY**		
No	109	(57.4%)
Yes	81	(42.3%)
**ROTTERDAM SCORE**		
1–4	135	(71.05%)
5–6	55	(28.95%)

All head CT scans performed by the patients included in this study were collected. The CT scans specifically evaluated for the purpose of this study were:

- the first CT scan on admission,
- the last CT scan achieved before DC (some patients performed only one CT scan before DC)
- the first CT scan performed after DC

Rotterdam score was calculated for each CT scan at admission. In each CT scan reported above we quantified the total volume and the side of each hemorrhagic contusions by measuring the ABC/2 volume (14) and summing the total hemorrhage volume of each contusion. We also evaluated the following variables on the initial head CT: cisternal effacement, midline shift >1 cm, subarachnoid hemorrhage, epidural hematoma, subdural hematoma, the number of hemorragic contusions pre-DC, and the total volume of contusions pre-DC.

Even surgical data including timing of DC and type of surgical approach (unilateral or bifrontal) were analyzed.

Finally, in the multivariate regression models the following variables were included:

- Age;
- GCS at admission;
- Pupils reactivity to the light;
- Timing of DC;
- Surgical approach (unilateral or bifrontal craniectomy);
- State of cisterns;
- Midline shift;
- Presence of SAH;
- Rotterdam Score;
- The presence of Epidural Hematoma;
- The presence of Subdural Hematoma;
- Number of contusions;
- Total Volume of Contusions;

We also analyzed the number of patients who required a new surgery for the progression of hemorrhagic contusions and the clinical outcome (secondary end-point of the study) according to the presence or not of the development or expansion of a hemorrhagic contusion with the Glasgow Outcome Scale (5 point GOS) at 6-month follow-up. Death, persistent vegetative state and severe disability were considered as unfavorable outcome (GOS 1–3) while GOS 4–5 (good recovery and moderate disability) as favorable outcome. The mean follow-up period was 18 months (minimum 6 months–maximum 5 years).

### Statistical Analyses

Data were analyzed with statistical package for social sciences (SPSS Inc., Chicago, Illinois, USA). Univariate analysis was performed by comparing patients who presented the growth or expansion of hemorrhagic contusion after DC in TBI and patients who did not. Continuous variables were compared using Student's *t* tests and Chi-square Test for discrete variables. The multiple logistic regression was used to identify independent risk factors. Power of the regression model to discriminate contusion progression was evaluated using receiver operating characteristic curve (ROC curves). Then a Pairwise *t* tests was used to compare the ROC curves. Statistical significance was set at *p* < 0.05.

## Results

The baseline characteristics of 190 patients treated with DC were summarized in Table 1.

Among these 190 patients, eight patients did not have any postoperative head CT scan and they were excluded. Then, 182 patients (149 males and 41 females) were finally included in this study.

Hemorrhagic contusions were detected on the initial CT scan or in the last CT scan before surgery in 103 out of 182 patients (56.6%). Among these, 31 patients presented only one contusion while the remaining 72 patients suffered from multiple hemorrhagic contusions. The mean volume of contusions at the initial CT scan was 12.8 ± 2.7 cc.

After DC, new or blossoming hemorrhagic contusions were observed in 47 patients out of 182 (25.82%). Among these, 40 patients presented an expansion of the hemorrhagic contusion already depicted in pre-DC CT scan with a mean volume of 39.1 ± 3.1 cc, while 7 patients developed new hemorrhagic contusion with a mean volume of 28.3 ± 1.4 cc. Among these patients, 11 out of 47 (23.4%) required a new surgical intervention for uncontrollable ICP after DC or for the onset of one dilated pupil.

The results of univariate analysis were summarized in Table 2: age was not significantly associated with the progression or the growth of a contusion, while patients with new or blossoming contusion after DC presented a significantly lower GCS than those who did not (*p* < 0.028). No significant difference in pupils' reactivity to light, timing of DC and surgical approach (unilateral or bifrontal DC) were present between patients with or without blossoming of contusion. Among radiological data (presence of subarachnoid hemorrhage SAH, state of cistern, midline shift, presence of subdural or epidural hematoma and Rotterdam Score), only the presence of subdural hematoma was significantly associated with new or expanded hemorrhagic contusions at univariate analysis (*p* = 0.027). Finally, among the 103 patients with hemorrhagic contusions at pre-DC CT scan, the presence of multiple hemorrhagic contusions and a total volume of contusions < 20 cc were significantly associated contusions progression (respectively *p* = 0.043 and *p* < 0.0001).

**Table 2:** Demographic, clinical, and imaging data for 182 patients included in statistical univariate analysis.

**Variabile**	**Total *N*°(%)**	**Contusion Expansion after DC**	
		**Yes *N*°(%)**	**No *N*°(%)**	***P*-Value**
Total number of patiens	182	47 (25.82%)	135 (74.18%)	
Age (years)				0.944
< 65 years	115 (63.2%)	29 (61.7%)	86 (63.7%)	
≥65 years	67 (36.8%)	18 (38.3%)	49 (36.3%)	
GCS at admission				**0.028**
3–5	126 (69.2%)	39 (82.9%)	87 (64.4%)	
6–8	56 (30.8%)	8 (17.1%)	48 (35.6%)	
Pupils reactivity to the light				0.578
Yes	148 (81.3%)	40 (85.1%)	108 (80%)	
No	34 (18.7%)	7 (14.9%)	27 (20%)	
Timing of DC				0.0771
< 48 h	149 (81.9%)	43 (91.4%)	106 (78.5%)	
≥48 h	33 (18.1%)	4 (8.6%)	29 (21.5%)	
Surgical approach				0.981
Unilateral	145(79.7%)	38 (80.85%)	107 (79.25%)	
Bifrontal	37(20.3%)	9 (19.15%)	28 (20.75%)	
Cisterns				0.65
Compressed	149 (81.9%)	40 (85.1%)	109 (80.75%)	
Absent	33 (18.1%)	7 (14.9%)	26 (19.25%)	
Midline Shift				0.953
>10 mm	133 (73.1%)	34 (72.34%)	99 (73.33%)	
< 10 mm	49 (26.9%)	14 (37.86%)	47 (26.77%)	
SAH				0.988
Yes	126 (69.2%)	33 (70.2%)	93 (68.8%)	
No	56 (30.8%)	14 (29.8%)	42 (31.2%)	
Rotterdam Score				0.947
1–4	98 (53.85%)	25 (53.1%)	73 (54%)	
5–6	84 (46.15%)	22 (46.9%)	62 (46%)	
Epidural Hematoma				0.96
Yes	17 (9.34%)	4 (8.5%)	13 (9.62%)	
No	165 (90.66%)	43 (91.5%)	122 (90.38%)	
Subdural Hematoma				**0.0188**
Yes	133 (73.1%)	41 (87.23%)	92 (68.14%)	
No	49 (26.9%)	6 (12.77%)	43 (31.86%)	
Number of contusions				**0.0436**
single	31 (30.1%)	12 (30%)	19 (30.15%)	
multiple	72 (69.9%)	28 (70%)	44 (69.85%)	
Total Volume of contusions				**<0.0001**
< 20 cc	68 (66.6%)	6 (15%)	62 (98.5 %)	
>20 cc	35 (33.4%)	34 (85%)	1 (1.5%)	

*N°(%) = number of patients (percentage of patients). Bold Values are the values statistically significative*.

At multivariate analysis, only the presence of an acute subdural hematoma (*p* = 0.0076) and a total volume of contusions >20 cc before DC (*p* = < 0.0001) were significantly associated with expansion of hemorrhagic contusions (Table 3).

**Table 3:** Multiple logistic regression model after variable selection (data is based on CT-scans before DC).

**Variable**	**OR**	**95% CI**	**Coefficient**	**Standard Error**	***P*-Value**
GCS at admission	1.9238	0.2537 to 14.5865	0.6543	1.0336	0.5267
Number of contusions	0.3144	0.3144 to 29.8745	−1.1572	1.0568	0.2735
Subdural Hematoma	**29.7859**	**2.4677 to 359.5208**	3.3940	1.2708	**0.0076**
Total Volume of contusions	**0.0012**	**0.0001 to 0.0112**	−6.7661	1.1624	**<0.0001**

*Bold Values are the values statistically significative*.

**Figure 1:**
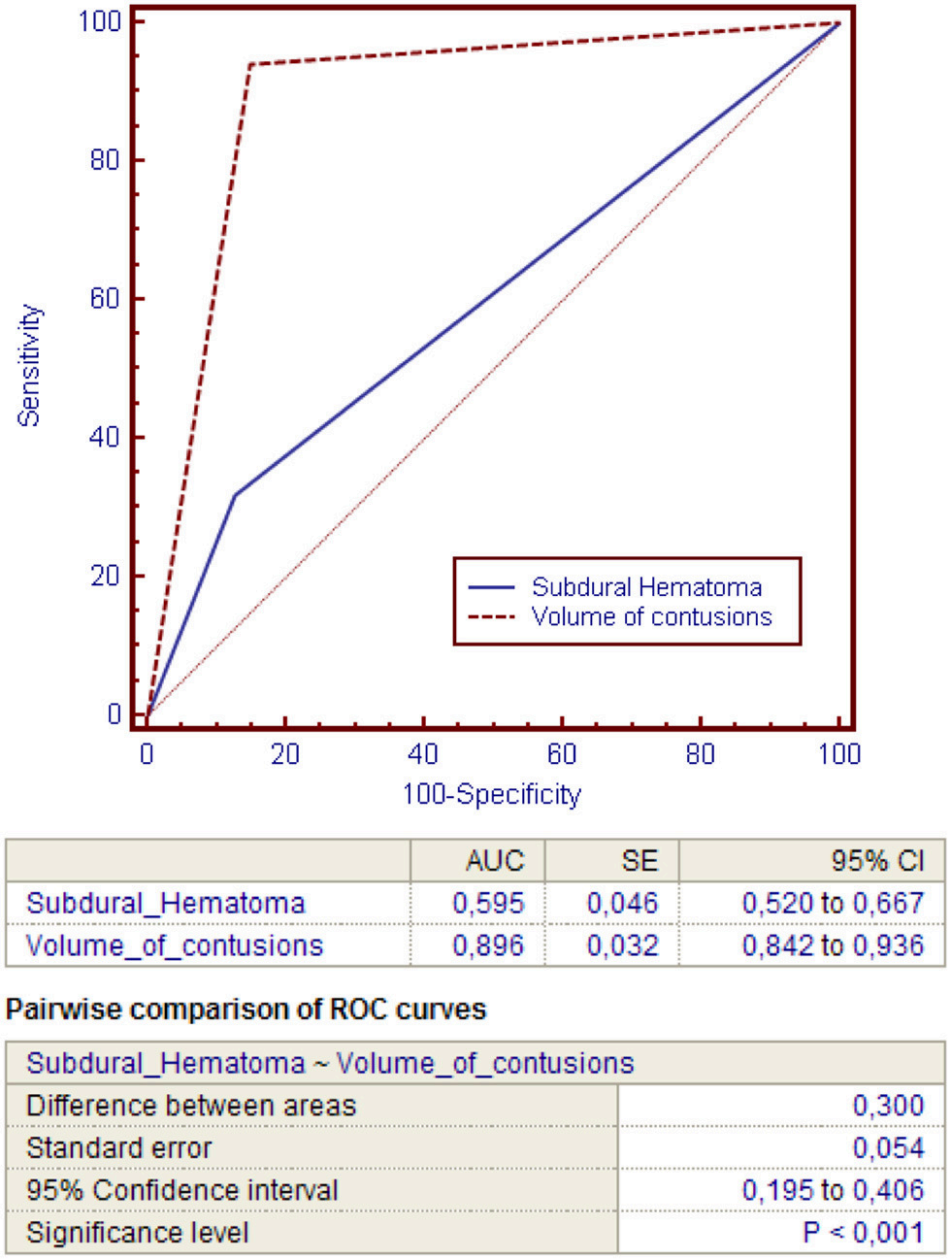
ROC curves analysis.

Then, power of the regression model to discriminate contusions progression was evaluated using ROC curves (Figure 1). The total volume of contusions before DC resulted to have higher accuracy and ability to predict postoperative blossoming of contusion with strong statistical significance rather than the presence of an acute subdural hematoma (these risk factors presented respectively an area under the curve [AUC] of 0.896 vs. 0.595; *P* < 0.00; Table 4).

**Table 4:** ROC curves analysis for comparing the accuracy of the presence of subdural hematoma or pre-DC total volume of contusions in predicting contusion expansion after DC.

**Variable**	**AUC**	**SE**	**95% CI**
Subdural Hematoma	0.595	0.046	0.52 to 0.667
**Total Volume of contusions**	0.896	0.032	0.842 to 0.936
**PAIRWISE COMPARISON OF ROC CURVES**			
Difference between area		0.300	
SE		0.054	
95% CI		0.195 to 0.406	
Significance level		*P* < 0.001	

*ROC, Receiver Operating Characteristic; AUC, Area Under the Curve; SE, Standard Error; CI, Confindence Interval*.

**Figure 2:**
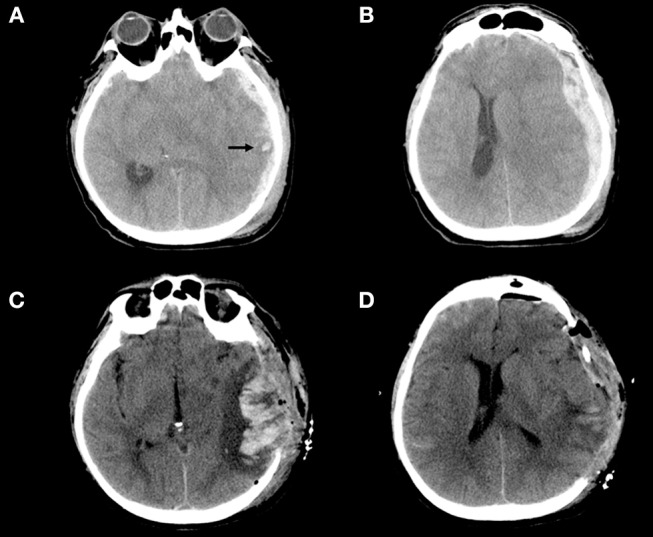
**(A–D)** Example of blossoming contusion in a 52-year-old male patient treated with DC for traumatic acute subdural hematoma. The patient sustained a close head injury in a motor vehicle accident and 2 h after trauma was admitted at our hospital with a Glasgow Coma Scale score of 6 (E1V1M4) and was put under mechanical ventilation. The pupils were bilaterally equal and reactive. **(A,B)** (Pre-operative CT scan). Pre-operative CT scan, immediately performed after admission of the patient described above, showing a large left acute subdural hematoma with a small temporal contusion [black arrow–**(A)**] and midline shift **(B)**; then the patient was immediately operated. **(C,D)** (Post-operative CT scan) Post-operative CT scan performed about 3 h after DC demonstrating massive contusion expansion **(C)** after wide DC **(D)**. During surgery bone flap was not replaced for massive brain swelling without evidence of bleeding/contusion enlargement at the level of temporal lobe.

**Figure 3:**
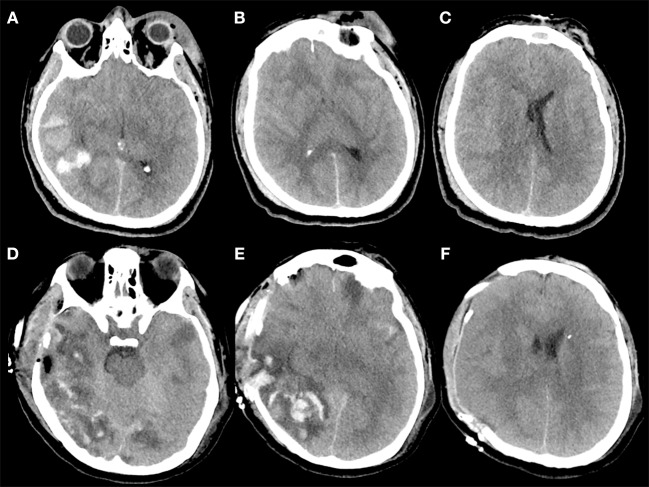
**(A–F)** Example of contusion expansion in a 35-year-old patient who underwent to DC for uncontrollable high ICP secondary to brain swelling and temporal hemorrhagic contusion with total volume > 20 cc. The patient sustained a polytrauma with TBI after intentional precipitation and 3 h after trauma was admitted at our hospital with a Glasgow Coma Scale score of 4 (E1V1M2) and was put under mechanical ventilation. The pupils were bilaterally equal and reactive. **(A–C)** (Pre-operative CT scan) Pre-operative CT scan, immediately performed after admission of the patient described above, showing a right temporal post-traumatic hemorrage with total volume > 20 cc **(A)**, diffuse brain swelling **(B)**, and left midline shift **(C)**; after this CT, an intraparenchymal probe for ICP monitoring was placed on the right side with stable values of ICP up to 30 mm Hg despite maximal medical treatment. Then the patient was immediately operated and a right DC was performed. During surgery, no active bleeding was observed at the level of temporal contusion and for this reason no lobectomy was performed. **(D–F)** (Post-operative CT scan) Post-operative CT scan performed 1 h after surgery demonstrating massive contusion expansion **(D,E)** and initial herniation of the brain away from the craniectomy defect **(F)**.

Indeed, the volume of contusions presented significant higher sensibility (94.07%) and specificity (85.11%) compared with the presence of acute subdural hematoma (respectively 31.85 and 87.23; Figures 2, 3).

Finally, the patients with blossoming hemorrhagic contusions after DC presented higher risk of unfavorable outcome compared with patients with no or stable contusions (*p* < 0.0185; Table 5).

**Table 5:** Outcome at 6-months follow-up of patients undergoing DC after severe TBI categorized by the presence or not of new or expansion of hemorrhagic contusion.

**Outcome**	**Total**	**Expansion of hemorrhagic contusions**		***P*-Value**
		**Yes**	**No**	
Unfavorable (GOS 1-3)	124 (68.2%)	39 (82.98 %)	85 (62.9%)	**<0.0185**
Favorable (GOS 4-5)	58 (31.8%)	8 (17.02%)	50 (37.1%)	

*Bold Values are the values statistically significative*.

## Discussion

Recent literature, including several retrospective studies and two recent randomized controlled trials (1–11), reported that while DC is able to reduce ICP and mortality of patients with uncontrollable ICP after TBI, on the other hand, it leads to an increase of patients with severe disability (1–11).

This data is related also to the high rate of complications secondary to DC (including hemorrhagic complications, infectious complications and disturbances of the CSF compartment) greater than any other neurosurgical intervention (12–22). The growth or expansion of hemorrhagic contusion is one of the main complications of DC after TBI (12–15).

For this reason, the identification of risk factors associated with new or worsened cerebral hemorrhage after DC is important in order to optimize diagnostic and management strategies. However, while the incidence and risk factors for blossoming contusions after TBI has been extensively analyzed, the same phenomenon following DC have not been thoroughly investigated (2, 12–15).

In a recent review about the complications of DC (12), the overall rate of “hemorrhagic complications” in TBI (including new or progression of epidural, subdural or intracerebral hemorrhage) was 11.9% and was secondary only to CSF disturbances (18.4%). Moreover, in this review hemorrhagic progression of a contusion was observed in 12.6% (163/1256) of TBI patients treated with DC. These authors reported that new and expanding hematomas occur early after DC, and they suggested as possible cause the loss of the tamponading effect of high ICP (12–14).

In our series, the rate of growth or expansion of hemorrhagic contusion after DC was higher than in the above-mentioned review (25.82 vs. 12.6%). However, this review (12) did not report the definition of each study included about the hemorrhagic contusion progression and this issue may underestimate the real incidence of this complication.

In this regard, Flint et al. (14) reported new or expanded hemorrhagic contusions ≥ 5 cc after DC in 23 patients out of 40 (58%).

Hemorrhagic contusion expansion was closely linked to the injury process after TBI and in previous studies it was observed in 42% of 142 TBI patients with median GCS scores of 8 and in 47% of 141 patients with traumatic subarachnoid hemorrhage (1, 2, 13). Furthermore, several studies identified DC as risk factor for contusion progression after TBI (23).

However, the real impact of DC on contusion expansion and the pathophysiology has not been clearly investigated in literature (12–15).

In a recently experimental animal model (about the effect of DC in a murine model of head injury), DC increases the devolvement of brain edema and contusions expansion (24). The authors postulated, as a possible pathophysiological explanation, that in their model a mechanic relief of tamponade effect due to DC may favor further increase of intracerebral bleeding with the beginning of vicious circle characterized by peri-hemorrage edema and secondary ischemic-hemorrhagic changes.

The main goal of this study was to identify the risk factors associated with the growth or expansion of hemorrhagic contusion after DC in TBI with the aim to identify a subgroup of decompressed who may benefit from closer CT monitoring and targeted therapies aimed at reducing intracranial bleeding.

Previous studies (14, 15) reported that the value of Rotterdam score, a radiological measure of TBI severity on the first CT scan, was associated with the blossoming contusions after DC. Flint et al. (14) reported that patients with Rotterdam scores 5 or 6 had an 78,6% (11/14) chance of expansion of their hemorrhagic contusions after DC.

On the contrary, in our study the Rotterdam score was not statistically associated with the growth or progression of the hemorrhagic contusion. Instead, in our series, the only radiological variable independently associated with increased risk of blossoming contusion was the presence of an acute subdural hematoma (*p* = 0.0076). In previous studies about contusion progression after DC this association was not found, however several papers focused on the natural history of brain contusion reported an independent association between acute subdural hematoma and contusion blossoming (24, 25). Alahmadi et al. (25) suggested (as possible explanation) that some acute subdural hematoma might be secondary to a burst lobe from an underlying large contusion that was more likely to enlarge.

Furthermore, Wang et al. (15) presented in their prediction model of the risk factors of hemorrhagic contusions a clear relationship between increased hemorrhage volumes and GCS score. This data was not confirmed by our study in where GCS score was similar in patients with and without hemorrhage expansion after DC.

The second factor in our study independently related to the development of new contusion or expansion of intracerebral post-traumatic hemorrhage, was the initial volume of brain contusion. Thirty Four patients out of 35 (97%) with a total hemorrhage volume > 20 cc at pre-DC CT scan presented expansion of contusion.

Neither Wang (15) nor Flint (14) have analyzed in their studies the association between the total volume of contusions before DC and the rate of progression of the contusions themselves. While in other studies about the natural history of post-traumatic hemorrhagic contusion among all baseline variables, the initial contusion volume represents the more accurate prognostic factor (2, 24). Several studies reported that the initial volume of hemorrhagic contusion was proportionally related to the rate of hemorrhagic progression, with smaller lesions remaining relatively stable and larger ones more likely to enlarge (24).

In our series, we evaluated the power of each independent variable at the regression model using ROC curves. The total volume of contusions resulted to have higher accuracy and ability to predict postoperative blossoming of contusion with strong statistical significance rather than the presence of acute subdural hematoma (these risk factors presented respectively an area under the curve [AUC] of 0.896 vs. 0.595; *P* < 0.001).

Indeed, the pre-DC total volume of contusions presented significant higher sensibility (94.07%) and specificity (85.11%) compared with the presence of acute subdural hematoma (respectively, 31.85 and 87.23%).

Finally, patients with new or expansion of hemorrhagic contusions after DC presented an increased risk for poor outcome (*p* < 0.0185).

The main limitations of this study included:

- The association between contusion blossoming and unfavorable outcomes at 6-month follow- up may be most likely due to their association with more severe primary brain injury and did not necessarily confirm a cause-and-effect relationship. Indeed, because the indication to DC was essentially based on clinical conditions, patients with the most severe TBI were those likely treated with DC.
- Another important issue is the eventuality that a significant rate of hemorrhagic contusion expansion might have occurred after the last pre-operative CT scan but before DC. This factor inserts a source of bias: it is not possible in fact to exclude in our study that a significant part of the hemorragic contusions evolved before the DC
- From our analysis, we excluded patients with antiplatelet or warfarin use or patients with coagulopathies already known before the trauma were excluded from the study. Data on blood tests on coagulation were not collected. This is fact involves another possible bias: we cannot exclude in our series that several blossoming contusions could be due to unknown coagulation disorders
- A mean of 5 CTs scans were performed for patient. Unfortunately, CT scans were not all analyzed on the same day of follow-up after DC; but they were analyzed as first, second and third CT after DC for each patient. This limitation may underestimate the effect of DC on the expansion of contusion in patients with early CT scan after surgery compared with patients with late CT scan.
- Finally, the manipulation of swollen and contused brain during the evacuation of acute subdural hematomas (ASDH) may lead in some cases to the development of new contusions or to the expansion of pre-existent traumatic intraparenchymal hemorrhage. Therefore, in our study the expansion of a part of contusions after ASDH evacuation could be secondary to a direct surgical complication rather than to an effect of the DC itself.

## Conclusions

The presence of an acute subdural hematoma was associated with an increasing rate of new or expanded hemorrhagic contusions after DC. The total volume of hemorrhagic contusions >20 cc before surgery was an independent and extremely accurate predictive sign of contusion blossoming in decompressed patients for severe TBI. Patients with blossoming contusions after DC presented an increased risk for unfavorable outcome.

## Ethics Statement

This study was carried out in accordance with the recommendations of name of guidelines, name of committee with written informed consent from all subjects. All subjects gave written informed consent in accordance with the Declaration of Helsinki. The protocol was approved by the name of committee.

## Author Contributions

DN: design of the work, analysis and interpretation of data for the work, and drafting the work. LdS: acquisition, analysis, and interpretation of data for the work. MG: acquisition, analysis, and interpretation of data for the work. EM: design of the work, acquisition, and interpretation of data for the work. MS: design of the work and revising the work critically. MI: interpretation of data for the work, and revising the work critically. MD: design of the work, drafting the work and revising the work critically. All authors: final approval for publication of the content, and agreement to be accountable for all aspects of the work in ensuring that questions related to the accuracy or integrity of any part of the work are appropriately investigated and resolved.

### Conflict of Interest Statement

The authors declare that the research was conducted in the absence of any commercial or financial relationships that could be construed as a potential conflict of interest.

## Abbreviations

DC: decompressive craniectomy
TBI: traumatic brain injury
SAH: subarachnoid hemorrhage
GCS: glasgow coma scale
GOS: glasgow outcome scale
Gd: gadolinium
DTI: diffusion tensor imaging
SWI: susceptibility-weighted imaging
VEI: vessel-enhanced images
TEI: tissue-enhanced images
3D PRESTO: three-dimensional principles of echo shifting with a train of observation
MinIP: minimum intensity projection
SWM: superficial white matter
DMV: deep medullary vein.
